# Denervation atrophy is independent from Akt and mTOR activation and is not rescued by myostatin inhibition

**DOI:** 10.1242/dmm.014126

**Published:** 2014-02-06

**Authors:** Elizabeth M. MacDonald, Eva Andres-Mateos, Rebeca Mejias, Jessica L. Simmers, Ruifa Mi, Jae-Sung Park, Stephanie Ying, Ahmet Hoke, Se-Jin Lee, Ronald D. Cohn

**Affiliations:** 1 McKusick-Nathans Institute of Genetic Medicine, The Johns Hopkins University School of Medicine, Baltimore, MD 21205, USA; 2 Department of Neurology, The Johns Hopkins University School of Medicine, Baltimore, MD 21205, USA; 3 Department of Molecular Biology and Genetics, The Johns Hopkins University School of Medicine, Baltimore, MD 21205, USA; 4 Department of Pediatrics, The Johns Hopkins University School of Medicine, Baltimore, MD 21205, USA; 5 Centre for Genetic Medicine, The Hospital for Sick Children, Toronto, ON M5G 1L7, Canada.

**Keywords:** Skeletal muscle, Muscle atrophy pathophysiology, TGF-β signaling, Myostatin, Denervation atrophy

## Abstract

The purpose of our study was to compare two acquired muscle atrophies and the use of myostatin inhibition for their treatment. Myostatin naturally inhibits skeletal muscle growth by binding to ActRIIB, a receptor on the cell surface of myofibers. Because blocking myostatin in an adult wild-type mouse induces profound muscle hypertrophy, we applied a soluble ActRIIB receptor to models of disuse (limb immobilization) and denervation (sciatic nerve resection) atrophy. We found that treatment of immobilized mice with ActRIIB prevented the loss of muscle mass observed in placebo-treated mice. Our results suggest that this protection from disuse atrophy is regulated by serum and glucocorticoid-induced kinase (SGK) rather than by Akt. Denervation atrophy, however, was not protected by ActRIIB treatment, yet resulted in an upregulation of the pro-growth factors Akt, SGK and components of the mTOR pathway. We then treated the denervated mice with the mTOR inhibitor rapamycin and found that, despite a reduction in mTOR activation, there is no alteration of the atrophy phenotype. Additionally, rapamycin prevented the denervation-induced upregulation of the mTORC2 substrates Akt and SGK. Thus, our studies show that denervation atrophy is not only independent from Akt, SGK and mTOR activation but also has a different underlying pathophysiological mechanism than disuse atrophy.

## INTRODUCTION

Healthy skeletal muscle maintains a balance between protein synthesis and protein degradation (Glass, 2010; McKinnell and Rudnicki, 2004). Disruption of this balance, resulting from a wide variety of conditions including immobilization (or disuse), chronic obstructive pulmonary disease, starvation, denervation or renal failure, can all lead to atrophy of the skeletal muscle (Glass, 2010; McKinnell and Rudnicki, 2004; Rüegg and Glass, 2011). Many studies have indicated that there is a common molecular pathway that is disrupted in all forms of skeletal muscle atrophy regardless of their etiology (Lecker and Goldberg, 2002; Lecker et al., 2004; Sacheck et al., 2007). This paradigm is thought to revolve mainly around the insulin-like growth factor (IGF) pathway and the serine-threonine kinase protein kinase B, commonly referred to as Akt (Glass, 2010; Rommel et al., 2001; Schiaffino and Mammucari, 2011). The Akt signaling cascade can regulate muscle mass by both inhibiting protein degradation and promoting protein synthesis; overexpression of Akt can lead to both muscle hypertrophy and the prevention of atrophy (Bodine et al., 2001b; Glass, 2010; Rommel et al., 2001).

Activated phosphorylated Akt (pAkt) blocks atrophy by phosphorylating and thus inactivating the transcription factor FoxO3a (Mammucari et al., 2007; Sandri et al., 2004; Zhao et al., 2008). The phosphorylated form of FoxO3a is excluded from the nucleus and therefore unable to activate the muscle-specific E3 ubiquitin ligases atrogin-1 and MuRF1, collectively referred to as atrogenes (Bodine et al., 2001a; Gomes et al., 2001; Mammucari et al., 2007; Zhao et al., 2008). Atrogenes have been shown to mediate the loss of muscle mass in multiple pathological conditions (Lecker and Goldberg, 2002; Lecker et al., 2004; Sacheck et al., 2007).

Akt is also capable of inducing hypertrophy by promoting protein synthesis through the activation of the mammalian target of rapamycin (mTOR) pathway (Bodine et al., 2001b; Glass, 2010; Laplante and Sabatini, 2012; Ma and Blenis, 2009). The mTOR pathway consists of two complexes, mTORC1 and mTORC2 (Laplante and Sabatini, 2012; Ma and Blenis, 2009). Both complexes, when activated, contain the phosphorylated form of mTOR and the shared scaffold protein mammalian lethal with Sec13 protein 8 (mLST8; also called GβL) (Inoki et al., 2005; Laplante and Sabatini, 2012; Ma and Blenis, 2009). The mTORC1 complex also includes the protein raptor and can phosphorylate eIF4E-binding protein (4E-BP1) and p70 ribosomal S6 kinase (p70S6k), both of which will lead to an increase in protein translation (Fenton and Gout, 2011; Ma and Blenis, 2009; Richter and Sonenberg, 2005). The phosphorylation and activation of p70S6k, specifically in skeletal muscle, has been shown to be dependent upon a second scaffold protein, eIF3f (Csibi et al., 2009; Csibi et al., 2008). The mTORC2 complex, with the unique protein rictor, creates a positive-feedback loop for both complexes by activating Akt and the serum and glucocorticoid-induced kinase (SGK) (Inoki et al., 2005; Laplante and Sabatini, 2012; Ma and Blenis, 2009). SGK has recently been shown to maintain and preserve skeletal muscle mass by inactivating FoxO3a and activating the mTOR pathway (Andres-Mateos et al., 2013; Aoyama et al., 2005; Brunet et al., 2001).

Myostatin is a protein that is thought to disrupt the balance between protein synthesis and protein degradation of healthy skeletal muscle by inhibiting Akt (Glass, 2010; Morissette et al., 2009; Sartori et al., 2009; Trendelenburg et al., 2009). Myostatin, also known as growth and differentiation factor-8 (GDF-8), is a naturally occurring potent negative regulator of skeletal muscle mass (Lee, 2004; Lee et al., 2005; McPherron and Lee, 1997). Mice deficient in myostatin and wild-type mice given a myostatin inhibitor both exhibit a profound hypertrophic muscle phenotype (Lee et al., 2005; McPherron and Lee, 1997). Myostatin is a member of the transforming growth factor-β (TGF-β) family of growth and differentiation factors (Lee, 2004; Lee et al., 2005). Myostatin and other TGF-β ligands will bind to cell-surface receptors and activate the canonical signaling cascade by the phosphorylation and activation of the Smad2/3 complex (MacDonald and Cohn, 2012; Rahimi and Leof, 2007). In addition, TGF-β ligands, including myostatin, are capable of activating several other non-canonical pathways, such as the phosphorylation and activation of the ERK1/2, p38 and JNK pathways (MacDonald and Cohn, 2012; Rahimi and Leof, 2007). The activation of both canonical and non-canonical TGF-β pathways can be detrimental to skeletal muscle and many studies have shown that inhibition of TGF-β signaling will ameliorate several types of myopathies (Burks et al., 2011; Cohn et al., 2007; Serrano et al., 2011).

**TRANSLATIONAL IMPACT**

**Clinical issue**

Healthy skeletal muscle maintains a balance between protein synthesis and protein degradation. Disruption of this balance from conditions such as inherited and acquired neuromuscular disorders can lead to atrophy of skeletal muscle. Myostatin (a member of the TGFβ family of growth and differentiation factors) naturally inhibits skeletal muscle growth. Consequently, the effects of myostatin inhibitors are being explored in animal models of inherited and acquired neuromuscular disorders and of age-related loss of muscle mass. The results from these studies have been mixed, with most benefits of myostatin inhibition being observed in the dystrophin-deficient *mdx* mouse, a model of inherited human muscular dystrophy. Although clinical trials of myostatin inhibition are being considered for individuals with muscular dystrophy, such patients would have to be treated throughout their lives and the risks associated with chronic treatment are currently unknown.

**Results**

To date, the use of myostatin inhibition for the treatment of acquired forms of myopathy, arising from immobilization (or disuse) or denervation has not been extensively studied. Here, therefore, the authors investigate the possible benefits of myostatin inhibition in two mouse models of acquired muscle atrophy: a hindlimb immobilization model (disuse atrophy) and a sciatic nerve resection model (denervation atrophy). The authors demonstrate that myostatin inhibition can protect mice from developing disuse atrophy but that myostatin inhibition has no effect on an atrophy resulting from the loss of the neuromuscular connection. Molecular analysis shows that myostatin does not modify the canonical TGFβ signaling pathway in either mouse model. Instead, non-canonical TGFβ signaling pathways are of greater importance in understanding the effect of myostatin inhibition. Notably, the authors also show that denervation atrophy is not affected by activation of pro-growth molecules that have been shown to benefit other forms of atrophy.

**Implications and future directions**

These preclinical data show that myostatin inhibition can prevent disuse atrophy but not muscle atrophy caused by denervation. Thus, for myostatin inhibition to be effective, an intact nerve-muscle conduction system must be present. This is essential information for future clinical applications of myostatin inhibition. Of equal importance, these findings provide new information about the molecular basis of disuse atrophy and of denervation atrophy. Specifically, although it is widely believed that all forms of skeletal muscle atrophy follow a similar molecular pattern, this work suggests that the mechanism of denervation atrophy is different to that of other forms of muscle wasting.

Because inhibition of myostatin produces such a profound effect on skeletal muscle, multiple studies have tested the use of these inhibitors to treat inherited muscle disorders. Myopathies such as dystrophin-negative muscular dystrophy, limb girdle muscular dystrophy and spinal muscular atrophy, among others, have all been treated with myostatin inhibitors (Morine et al., 2010; Morrison et al., 2009; Ohsawa et al., 2006; Sumner et al., 2009; Wagner et al., 2002). However, only a few studies have attempted to use myostatin inhibitors for the treatment of acquired myopathies and they have focused mainly on systemic conditions such as cancer cachexia, diabetes, or even obesity (Guo et al., 2012; Guo et al., 2009; Koncarevic et al., 2012; Zhou et al., 2010).

In our study we wanted to test the hypothesis the soluble ActRIIB receptor, a myostatin receptor fused to an Fc domain (Lee et al., 2005) that inhibits myostatin signaling, is able to prevent single-limb, acquired muscle atrophy. We used two mouse models for this purpose: a hindlimb immobilization model (disuse atrophy) and a sciatic nerve resection model (denervation atrophy). Our results indicate that myostatin inhibition is beneficial in settings of disuse, but not denervation, atrophy. Our subsequent molecular analysis and comparison of these two atrophy models led us to the surprising conclusion that denervation atrophy is not dependent upon the activation of Akt, SGK or mTOR, suggesting that there is not a universal pathway responsible for all forms of atrophy and therefore denervation should be treated as a distinct pathogenic condition.

## RESULTS

### Myostatin inhibitor ActRIIB protects muscle from disuse, but not denervation, atrophy

**Fig. 1.**
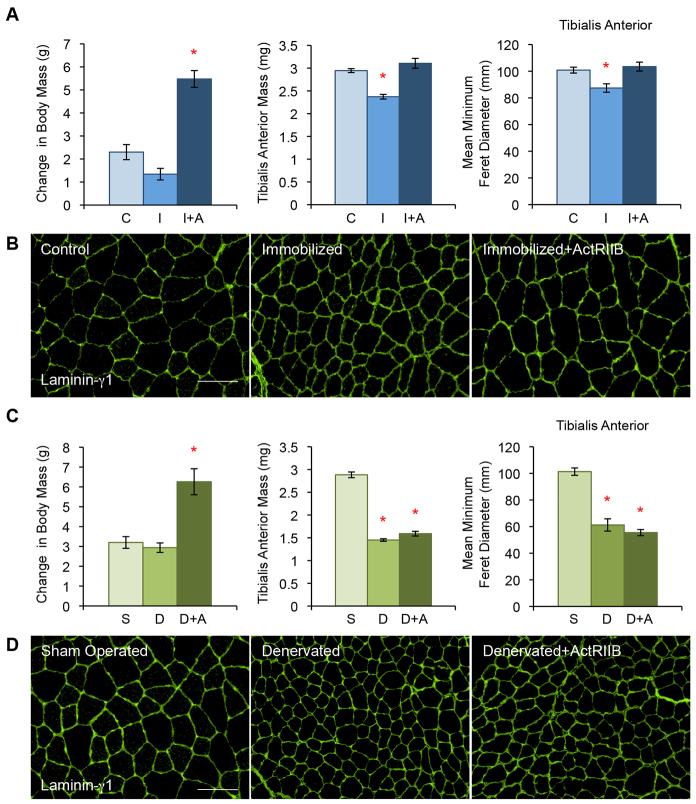
**Myostatin inhibition prevents disuse, but not denervation, atrophy.** (A,C) ActRIIB treatment leads to an increase in body mass for both the immobilization (‘I+A’) (*P<5.0×10^−8^) and denervation (‘D+A’) (*P<1.0×10^−4^) models (panels A and C, respectively, left graph). (A) The TA mass of immobilized (‘I’) mice is significantly lower than controls (‘C’) (*P<1.0×10^−8^); however, no loss of muscle mass is seen in ActRIIB-treated immobilized mice (center graph). MFD quantification of the fiber size of immobilized TA muscle (A, right graph) confirmed visual analysis by laminin-γ1 staining (B) that the immobilized mice lose fiber size (*P<1.0×10^−2^) but not when treated with ActRIIB. (C) The denervated TA muscle (‘D’) is significantly smaller than sham-operated controls (‘S’) (*P<5.0×10^−15^) and this is not prevented by ActRIIB treatment (center graph). MFD quantification (C, right graph) and visual analysis (D) showed that both the denervated and denervated with ActRIIB treatment lose the same amount of muscle fiber diameter over the course of the treatment (*P<1.0×10^−4^). Data are represented as mean ±s.e.m. *P-values indicate significant differences with respect to controls. Scale bars: 100 μm.

To assess whether myostatin inhibition would protect muscle from atrophy, we used two separate mouse models. We either attached a surgical staple to immobilize one hindlimb of our mice or denervated them by surgical removal of the sciatic nerve from one hindlimb, and then treated both groups with 10 mg/kg ActRIIB for 3 weeks. Owing to the enlargement of all non-challenged muscle, ActRIIB treatment resulted in a substantial increase in total body mass in both atrophy models (Fig. 1A,C, left graphs).

The immobilized placebo-treated group had 19.5% less tibialis anterior (TA) muscle mass compared with controls (Fig. 1A, center graph). However, the ActRIIB-treated immobilized mice did not show loss of TA muscle mass compared with untreated controls (Fig. 1A, center graph). When compared with controls, the measurement of the minimum feret diameter (MFD) showed a similar pattern of loss of muscle fiber diameter in the immobilized placebo group (13.3% reduction), but not in the ActRIIB-treated mice (Fig. 1A, right graph, 1B).

In contrast to the immobilization experiment, both the placebo- and ActRIIB-treated denervated mice lost a significant amount of TA muscle mass compared with sham-operated controls (49.7% and 44.8%, respectively; Fig. 1C, center graph). Measurement of the MFD also showed that the denervated groups had 39.6% and 45.2% smaller muscle fibers (placebo- and ActRIIB-treated, respectively) compared with sham-operated controls (Fig. 1C, right graph, 1D).

Based on muscle-mass and fiber-size measurements, we found that myostatin inhibition protects against disuse, but not denervation, atrophy.

### Non-canonical TGF-β signaling markers are targeted by ActRIIB treatment

**Fig. 2.**
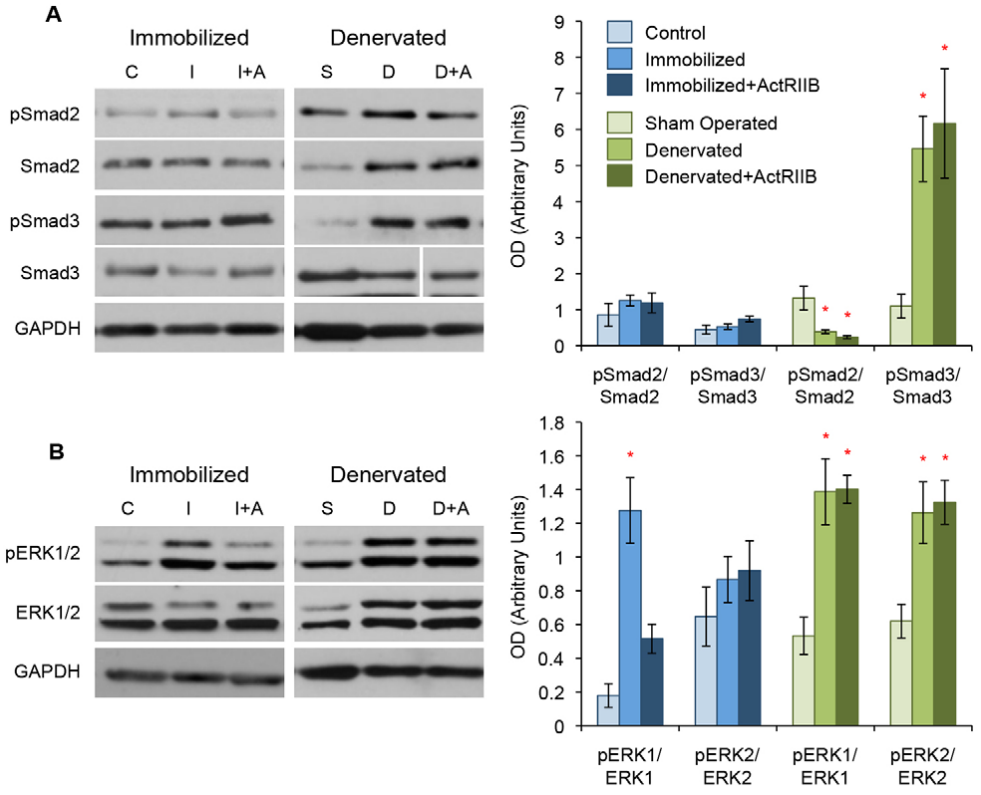
**ActRIIB treatment targets non-canonical TGF-β signaling markers in disuse atrophy.** Western blot analysis of TA muscle protein lysates. (A) Immobilization alone (‘I’) or with ActRIIB treatment (‘I+A’) did not show any difference in pSmad2 or pSmad3 levels compared with controls (‘C’). Denervation alone (‘D’) induced a significant increase in total Smad2 and active pSmad3 compared with sham-operated controls (‘S’); however, those changes are not reduced by ActRIIB treatment (‘D+A’). (B) Both immobilization and denervation resulted in an upregulation in active pERK1/2. ActRIIB treatment prevented the upregulation of pERK1/2 in immobilized but not denervated muscle. Quantitative analysis of blots is displayed in the graphs (right) with arbitrary units of mean ± s.e.m. *P<5.0×10^−2^ with respect to controls. Lines indicate where intervening lanes have been removed from a single image to show the most representative band for that treatment group.

To understand the molecular basis for the differences in ActRIIB treatment outcome in the immobilization and denervation models, we next performed western blot analysis of TA muscle protein lysates. We found that immobilization alone does not change the activation of the canonical TGF-β signaling markers Smad2 and Smad3. In addition, ActRIIB treatment of immobilized mice also did not alter the activation of Smad2 or Smad3 (Fig. 2A). Denervation alone induced a threefold upregulation in total Smad2 and a fivefold increase in active pSmad3, but ActRIIB treatment did not attenuate either of these (Fig. 2A). We subsequently examined the expression of the non-canonical TGF-β signaling markers extracellular-signal-regulated kinases 1 and 2 (ERK1/2). Both models of atrophy demonstrated an increase in active pERK1/2 compared with their respective controls (Fig. 2B). ActRIIB treatment prevented the activation of ERK in the immobilized model but not in the denervated model. Denervation also resulted in a significant upregulation of total ERK1/2 protein expression, something not observed in the immobilized model (Fig. 2B).

Myostatin also influences the expression of other proteins involved in muscle growth and regeneration, including myogenin and p21. Myostatin promotes the expression of p21 and inhibits the expression of myogenin, both of which will negatively regulate the differentiation of muscle precursor cells and therefore muscle growth (Langley et al., 2002; Ohsawa et al., 2006). In both models of atrophy, we found that p21 expression is unchanged, both with and without ActRIIB treatment. However, loss of myogenin was observed in the immobilization model but not in the immobilized treated with ActRIIB. Denervation alone induced a significant upregulation in myogenin, but was not changed further by ActRIIB treatment (supplementary material Fig. S1).

We found that muscle disuse results in an upregulation of the non-canonical TGF-β signaling marker pERK1/2, which was prevented by ActRIIB treatment. In addition, another marker of myostatin signaling, myogenin, is sensitive to immobilization but not in ActRIIB-treated mice. ActRIIB treatment did not inhibit the upregulation of canonical or non-canonical TGF-β signaling markers or any other marker of myostatin signaling in denervated muscle.

### Akt and SGK are dysregulated in disuse and denervation atrophy

**Fig. 3.**
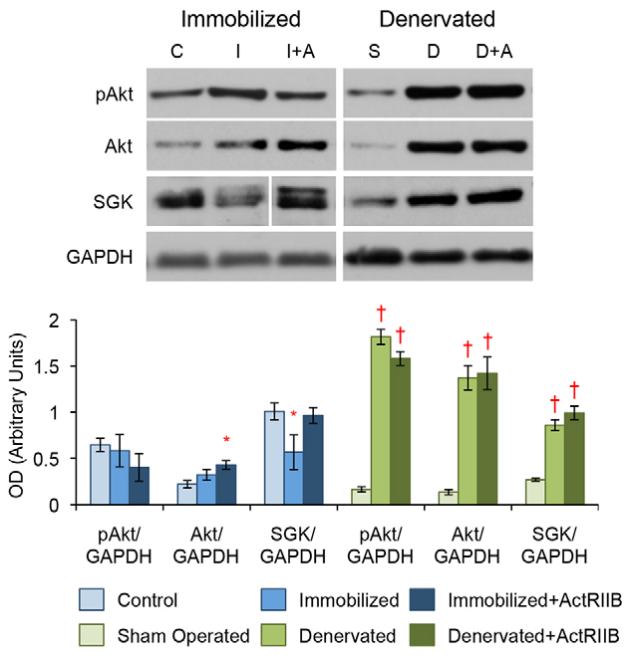
**Loss of SGK, but not Akt, is observed in disuse atrophy, but upregulation of both occurs in denervation atrophy.** Western blot analysis of TA muscle protein lysates. No change in active pAkt is seen between the control (‘C’), immobilized (‘I’) and immobilized with ActRIIB treatment (‘I+A’) groups. Total Akt is increased in immobilized mice treated with ActRIIB compared with controls. SGK expression is decreased with immobilization, but not when mice are treated with ActRIIB. Both denervation alone (‘D’) and with ActRIIB treatment (‘D+A’) lead to a substantial upregulation in active pAkt, total Akt and SGK when compared with sham-operated controls (‘S’). Quantitative analysis of blots is displayed in the graph (below) with arbitrary units of mean ± s.e.m. *P<5.0×10^−2^ and ^†^*P*<5.0×10^−5^ with respect to controls. Lines indicate where intervening lanes have been removed from a single image to show the most representative band for that treatment group.

Next we examined the expression levels of Akt in both atrophy models. We found that immobilization with or without ActRIIB treatment did not alter the amount of active pAKT (Fig. 3). An upregulation in total Akt, however, was observed in immobilized mice treated with ActRIIB compared with controls. Conversely, in both the placebo- and ActRIIB-treated denervated mice we found an 11-fold upregulation in phosphorylated and total Akt (Fig. 3).

Because the changes observed in Akt activation and expression cannot explain either the denervation or immobilization phenotypes, we also examined an additional regulator of muscle mass maintenance, SGK. We found that SGK expression was reduced almost twofold in immobilized mice, but not in immobilized mice treated with ActRIIB (Fig. 3). In contrast, both the placebo- and ActRIIB-treated denervated models showed a threefold upregulation of SGK expression when compared with sham-operated controls (Fig. 3).

**Fig. 4.**
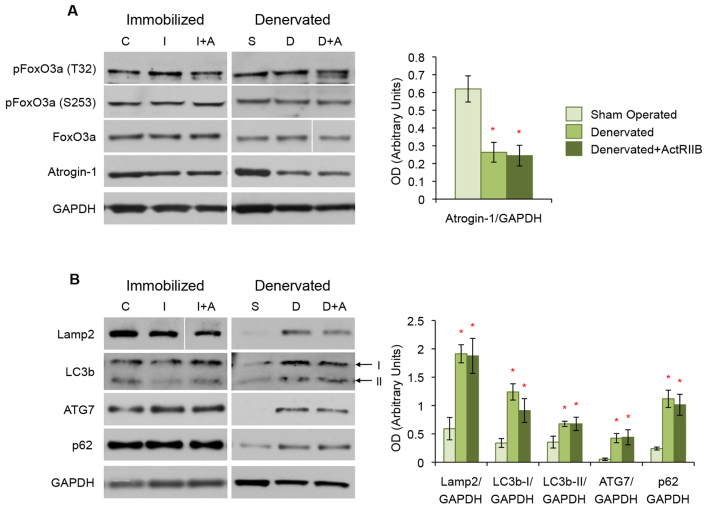
**Autophagy plays a significant role in denervation, but not disuse, atrophy.** Western blot analysis of TA muscle protein lysates. (A) Phosphorylation and total expression of FoxO3a did not change between the control (‘C’), immobilized (‘I’) and immobilized with ActRIIB treatment (‘I+A’) groups. In addition, with or without ActRIIB treatment, immobilization did not change the expression of the FoxO3a target atrogin-1. Similarly, no change was seen between the sham-operated (‘S’), denervated (‘D’) and ActRIIB-treated denervated (‘D+A’) mice in FoxO3a phosphorylation or total expression. Denervated and denervated with ActRIIB mice did, however, show a decrease in atrogin-1 levels compared with sham-operated controls. (B) Markers of autophagy were not different in placebo- and ActRIIB-treated immobilized mice when compared with controls. Denervation alone resulted in an upregulation in Lamp2, LC3b, ATG7 and p62 that was not changed further by ActRIIB treatment. Quantitative analysis of blots is displayed in the graphs (right) with arbitrary units of mean ± s.e.m. *P<5.0×10^−2^ with respect to controls. Lines indicate where intervening lanes have been removed from a single image to show the most representative band for that treatment group.

We next examined the expression and activation levels of FoxO3a, a downstream target of SGK and Akt. Both SGK and Akt have been shown to have equal affinity to phosphorylate FoxO3a at T32, but Akt preferentially phosphorylates S253, the site that will inactivate the protein (Brunet et al., 2001). In both atrophy models, with or without ActRIIB treatment, we found no significant difference in phosphorylation at either the T32 or S253 site or in total expression levels of FoxO3a (Fig. 4A). Expression levels of the FoxO3a target atrogin-1 further substantiated these results. We found no difference in expression in either immobilization model, but both placebo- and ActRIIB-treated denervated muscle had reduced atrogin-1 levels (Fig. 4A).

The Akt-mTOR-FoxO3a pathway is also known to play an important part in the regulation of autophagy in skeletal muscle (Schiaffino and Mammucari, 2011; Zhao et al., 2008). Examination of autophagy markers in immobilized mice showed no change between the control, placebo-treated and ActRIIB-treated groups (Fig. 4B). Denervation, however, induced a significant increase in several markers of autophagy, including Lamp2, LC3b, ATG7 and p62 (Fig. 4B). Treatment with ActRIIB did not further change the expression of autophagy markers in denervated muscle.

Our results showed that SGK, not Akt, is lost as a result of immobilization, but not when the muscle is protected by treatment with ActRIIB. Levels of both SGK and Akt were significantly upregulated in denervated muscle with or without ActRIIB treatment. Moreover, a significant upregulation in autophagy markers was observed in denervation, but not disuse, atrophy.

### The mTOR signaling pathway is upregulated in denervation atrophy

The mTOR signaling cascade plays an important role in skeletal muscle maintenance by promoting protein synthesis (Glass, 2010; Ma and Blenis, 2009). We therefore examined the activation of both the mTORC1 and mTORC2 complexes in our models of atrophy and ActRIIB treatment.

**Fig. 5.**
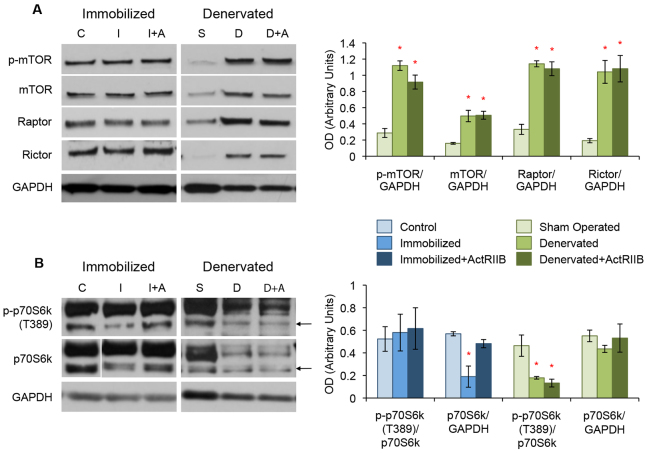
**Dysregulation of mTOR signaling in both disuse and denervation atrophy.** Western blot analysis of TA muscle protein lysates. (A) Expression levels of the components of the mTOR complexes, including p-mTOR, total mTOR, raptor and rictor, were not different between control (‘C’), immobilized (‘I’) and ActRIIB-treated immobilized (‘I+A’) mice. Denervation (‘D’) and denervation with ActRIIB treatment (‘D+A’) led to a substantial increase in p-mTOR, total mTOR, raptor and rictor when compared with sham-operated controls (‘S’). (B) Immobilization resulted in a decrease of p70S6k expression but not when the mice were treated with ActRIIB. Denervation led to a decrease in active phosphorylation of p70S6k with no loss of total protein expression and this was not prevented by treatment with ActRIIB. Quantitative analysis of blots is displayed in the graphs (right) with arbitrary units of mean ± s.e.m. *P<5.0×10^−2^ with respect to controls. Lines indicate where intervening lanes have been removed from a single image to show the most representative band for that treatment group. Arrows in B indicate the correct size of p70S6k.

We found no change in the mTOR complex components p-mTOR, total mTOR, raptor and rictor in immobilized mice with or without ActRIIB treatment (Fig. 5A). However, a scaffold protein common between both mTOR complexes, GβL, was downregulated with immobilization, but was maintained at control levels in ActRIIB-treated immobilized mice (supplementary material Fig. S2). A substrate of mTORC1, p70S6k, and a second scaffold protein, eIF3f, which facilitates the interaction between mTORC1 and p70S6k, also showed reduced expression in immobilized muscle (Fig. 5B and supplementary material Fig. S2). The loss of p70S6k and eIF3f expression was not seen in immobilized mice treated with ActRIIB (Fig. 5B). A second mTORC1 substrate, 4E-BP1, did not show any change in phosphorylation or total abundance in placebo-or ActRIIB-treated immobilized mice compared with controls (supplementary material Fig. S2).

The denervated mice demonstrated a significant upregulation in several components of both the mTORC1 and mTORC2 complexes (Fig. 5A). The three- to fivefold upregulation of p-mTOR, total mTOR, raptor and rictor was unchanged by ActRIIB treatment of denervated mice (Fig. 5A). The scaffold proteins GβL and eIF3F were also upregulated in both denervation models compared with sham-operated controls (supplementary material Fig. S2). Downstream of the mTORC1 complex, however, we found that denervation results in a significant, almost threefold, drop in phosphorylation at the T389 activation site of p70S6k, despite no change in total p70S6k expression (Fig. 5B). ActRIIB treatment of denervated mice did not prevent the loss of p70S6k activation. Both placebo and ActRIIB treatment of denervated mice led to an upregulation in total and phosphorylated 4E-BP1 (supplementary material Fig. S2).

Active p-mTOR can also regulate autophagy by the phosphorylation and inactivation of ULK1 (the mammalian homolog of *Caenorhabditis elegans* ATG1), a protein known to negatively regulate p70S6k (Egan et al., 2011; Lee et al., 2007). We found no change in total ULK1 expression or phosphorylation at the mTOR-specific inhibitory S757 site in immobilized mice, with or without ActRIIB treatment, compared with controls (supplementary material Fig. S3). Similarly, sham-operated, denervated and ActRIIB-treated denervated muscle did not show any difference in total ULK1 expression levels. However, both placebo- and ActRIIB-treated denervated mice did produce a significant drop in phosphorylation at the S757 inhibitory site compared with sham-operated controls (supplementary material Fig. S3).

We found that, compared with controls, muscle disuse did not change the levels of the main mTOR-complex components. However, immobilization did lead to reduced expression of p70S6k and several mTOR-associated scaffold proteins. Immobilized mice were protected from the loss of these proteins by treatment with ActRIIB. In contrast, denervation atrophy resulted in a significant upregulation in nearly all components of the mTOR pathway except for p70S6k – changes that were not prevented by ActRIIB treatment. Although total p70S6k expression did not change, denervation led to the loss of active p-p70S6k, which might be due to an increase in active ULK1.

### Rapamycin treatment does not alter the denervation atrophy phenotype

The upregulation of mTOR signaling observed in denervated muscle could be a compensatory mechanism employed to prevent further atrophy or could be contributing to the pathogenic phenotype (Ramos et al., 2012). In order to clarify this difference in our model, we next treated the denervated mice with the mTOR inhibitor rapamycin (2 mg/kg body weight) for 3 weeks.

**Fig. 6.**
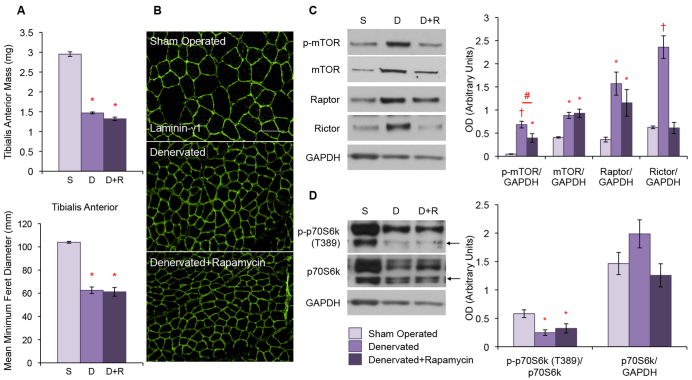
**Rapamycin treatment does not change the denervation atrophy phenotype but does reduce mTOR activation.** Denervation alone (‘D’) and with rapamycin treatment (‘D+R’) resulted in a significant loss of TA mass when compared with sham-operated controls (‘S’) (*P<5.0×10^−12^) (A; top graph). MFD analysis (A; bottom graph) and laminin-γ1 staining (B) showed that placebo- and rapamycin-treated denervated muscle both had significantly reduced muscle fiber diameter (*P<1.0×10^−6^). Scale bar: 100 μm. (C) Western blot analysis of TA muscle protein lysates. Denervation alone resulted in an upregulation of p-mTOR, total mTOR, raptor and rictor. Denervated mice treated with rapamycin also showed an upregulation of mTOR and p-mTOR; however, the upregulation of p-mTOR was significantly less than placebo-treated mice (#*P*<5.0×10^−2^). There was a trend towards reduced expression of raptor with rapamycin treatment in denervated muscle. Rictor levels were not elevated in denervated mice treated with rapamycin compared with sham-operated controls. (D) Loss of the active phosphorylation of p70S6k was seen in placebo- and rapamycin-treated denervated mice despite there being no change in total p70S6k expression. Quantitative analysis of blots is displayed in the graphs (right) with arbitrary units of mean ± s.e.m. For western blots, *P<5.0×10^−2^ and ^†^ *P*<5.0×10^−4^. All *P*-values indicate a difference with respect to controls unless otherwise noted.

We found that, compared with sham-operated controls, denervation resulted in a 50.2% loss of TA muscle mass that was not prevented by treatment with rapamycin (Fig. 6A, top graph). MFD measurements also showed that muscle fiber size was reduced by 39.8% and 41.1% in denervated muscle with or without rapamycin treatment, respectively, compared with sham-operated controls (Fig. 6A, bottom graph, 6B).

We then looked at the mTOR signaling pathway in TA muscle protein lysates. As previously demonstrated, muscle denervation led to an increase in p-mTOR, total mTOR, raptor, rictor, GβL and eIF3f compared with sham-operated controls (Fig. 6C and supplementary material Fig. S4). However, compared with the placebo group, rapamycin treatment of denervated mice resulted in a nearly twofold reduction in active p-mTOR. A trend towards reduced raptor expression was also seen in rapamycin compared with placebo-treated denervated mice, but it did not reach significance (Fig. 6B). We observed that, of the scaffold proteins, rapamycin treatment of denervated mice reduced the expression of GβL, but not eIF3f (supplementary material Fig. S4). No change was seen between the denervated and denervated with rapamycin treatment in the mTORC1 substrates p70S6k and 4E-BP1 (Fig. 6D and supplementary material Fig. S4). We also noted that the expression of rictor was not upregulated in denervated mice given rapamycin treatment, compared with sham-operated controls (Fig. 6B). A complete knock down of p-mTOR was achieved by treating denervated mice with a higher dose of rapamycin (10 mg/kg body weight); however, because the phenotype remained the same as with the low-dose treatment, we continued our analysis using the more physiological dose of 2 mg/kg body weight (supplementary material Fig. S5).

**Fig. 7.**
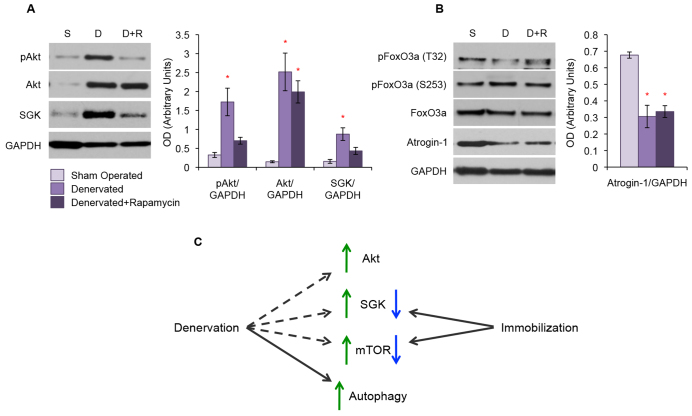
**Rapamycin treatment prevents upregulation of Akt and SGK in denervated muscle.** Western blot analysis of TA muscle protein lysates. (A) Denervation (‘D’) resulted in the upregulation of active pAkt, total Akt and SGK when compared with sham-operated controls (‘S’). The upregulation of active pAkt and SGK was not seen in denervated mice treated with rapamycin (‘D+R’). (B) Phosphorylation and expression of FoxO3a was unchanged between the denervation treatment groups. Denervation resulted in a decrease in atrogin-1 expression and this was not changed by rapamycin treatment. Quantitative analysis of blots is displayed in the graphs (right) with arbitrary units of mean ± s.e.m. *P<5.0×10^−3^ with respect to controls. (C) Diagram showing markers that are altered due to either disuse or denervation atrophy. Dashed lines indicate that denervation atrophy results in increased activation of these markers, yet they do not contribute to the pathological phenotype.

Because both Akt and SGK are substrates of the mTORC2 complex, we also examined the expression levels of these proteins in our rapamycin-treated denervation model. Denervation alone resulted in an upregulation of pAkt, total Akt, and SGK (Fig. 7A). However, we found that rapamycin treatment prevented the increase in active pAkt and SGK expression, but not the increase in total Akt expression, caused by denervation alone (Fig. 7A). We once again observed no difference in expression or activation of FoxO3a in denervated muscle, with or without rapamycin treatment. In addition, rapamycin treatment did not prevent the loss of atrogin-1 expression observed with denervation alone (Fig. 7B).

Our data demonstrate that rapamycin treatment does not rescue denervation atrophy despite inhibiting the activation of mTOR in the atrophic muscle. In addition, rapamycin treatment prevented the upregulation of pAkt and SGK – but not the downregulation of atrogin-1 – that is normally seen in denervation atrophy.

## DISCUSSION

The ability to prevent or treat acquired forms of skeletal muscle atrophy has the potential for wide-reaching benefits to millions of patients. Immobilization alone is a natural complication from many primary conditions, including limb casting, reduced movement when ill or bed rest, all of which can lead to atrophy of the skeletal muscles. The loss of muscle mass can prolong recovery from the primary condition of the patient and increase rehabilitation time. Myostatin inhibitors are excellent candidates for the treatment of acquired muscle atrophies owing to their dramatic and immediate effect on muscle. Indeed, numerous pharmaceutical companies are, in fact, currently working on developing myostatin inhibitors for the treatment of a variety of muscle disorders. A careful analysis of how these inhibitors influence muscle mass under various and unique pathological conditions is an essential step towards bringing them to the clinic.

Our studies show that myostatin inhibition has the potential for clinical application in the prevention of disuse atrophy. This protection was demonstrated by the preservation of both muscle mass and fiber diameter in immobilized mice treated with ActRIIB (Fig. 1A). However, when this type of treatment does advance to the clinic, it will have to be taken into consideration that currently available myostatin inhibitors cause an increase in mass of all skeletal muscles in the body. Of equal importance are our findings that myostatin inhibition is not effective against atrophy when the neuromuscular connection has been lost (Fig. 1C). This result was somewhat surprising given that the mouse model for amyotrophic lateral sclerosis (ALS), a disease that results in loss of muscle innervation, showed improvement with myostatin inhibition (Morrison et al., 2009). In light of our results, however, this phenomenon is probably due to the heterogeneity of the innervated and denervated muscle fibers that are a consequence of this disease. The innervated muscles would benefit and become larger as a result of myostatin inhibition and be able to compensate for the unaffected, non-innervated fibers. In addition, despite previous research suggesting that denervation leads to an increase in myostatin transcript and protein expression, our data indicate that this might not be the main reason for the loss of muscle mass (Baumann et al., 2003; Liu et al., 2007; Shao et al., 2007). Owing to the extensive post-translational processing and modification that occurs to myostatin before it is a mature protein, it is possible that the previous studies have measured non-functional protein or untranslated mRNA (Lee, 2004).

Our subsequent analyses of the TGF-β signaling pathway in skeletal muscle from the immobilized and denervated groups showed that myostatin inhibition did not reduced the canonical TGF-β signaling markers, pSmad2 and pSmad3, in either model (Fig. 2A). This is in contrast to previously published work demonstrating that myostatin itself will increase Smad activation in skeletal muscle and one report showing that ActRIIB will lower pSmad2 levels in a non-wild-type mouse (Langley et al., 2002; Rebbapragada et al., 2003; Sartori et al., 2009; Zhou et al., 2010). Our data might instead suggest that it is the downregulation of non-canonical TGF-β signaling markers that is of greater importance in understanding the effect that ActRIIB has on the muscle. However, it is possible that, owing to the long duration of our experiment, we have missed the ActRIIB-induced downregulation of Smad activation in our models.

We also explored the Akt-FoxO3a-mTOR pathway because myostatin itself can inhibit its activation and it is another commonly investigated atrophy regulation pathway (Glass, 2010; Trendelenburg et al., 2009). Neither of our atrophy mouse models, however, demonstrated the expected increase in activation and phosphorylation of Akt when treated with ActRIIB (Fig. 3) (Morissette et al., 2009; Trendelenburg et al., 2009). This complements a previous study showing that loss of Akt expression does not attenuate the muscle hypertrophy response to ActRIIB treatment (Goncalves et al., 2010). In addition, in our immobilization model, we found that SGK, not Akt, exhibited the expected pattern of loss of expression with atrophy but not in mice protected by ActRIIB treatment (Fig. 3). Our results suggest that loss of SGK could mediate long-term disuse atrophy and further supports our previous work showing that overexpression of SGK preserves muscle (Andres-Mateos et al., 2013). One speculation, which would reconcile our study with the previously mentioned reports of Akt-mediated regulation of disuse atrophy, is the idea of a temporal switch from Akt to SGK as the atrophy progresses. Our denervated model, on the other hand, produced the initially puzzling result that active pAkt, total Akt and SGK were all significantly upregulated in the atrophic muscle (Fig. 3). These results would have been expected in a hypertrophic muscle rather than in a severely diseased muscle phenotype. We initially hypothesized that the increase of phosphorylated Akt is of compensatory nature to prevent further exaggeration of muscle atrophy in response to denervation. However, the significant decrease of phosphorylated Akt signaling in denervated mice treated with rapamycin, despite no exacerbation of muscle atrophy, makes this less likely. We are currently exploring a number of different experiments to analyze this interesting observation.

We then proceeded to examine the downstream markers of Akt and SGK signaling to further clarify our results. We first examined the expression and activation of FoxO3a, the main transcription factor needed for the activation of atrogenes, a set of E3 ubiquitin ligases known to be highly involved in muscle atrophy (Lecker and Goldberg, 2002; Zhao et al., 2008). Despite loss or overexpression of either Akt or SGK, we found no difference in the phosphorylation of FoxO3a in either atrophy model (Fig. 4A). A FoxO3a atrogene target, atrogin-1, is also unchanged in the immobilized mice and is actually decreased in denervated muscle. We conclude that, at 3 weeks, neither FoxO3a nor atrogin-1 are mediating either form of atrophy or providing protection from disuse atrophy with ActRIIB treatment. It is possible that the involvement of FoxO3a and atrogen-1 are more immediate and occur at an earlier time point than we analyzed. A temporal involvement of atrogin-1 is indeed likely because an atrogin-1 knockout mouse is partially protected from denervation atrophy (Bodine et al., 2001a).

We also examined markers of another type of protein degradation regulated by the Akt-mTOR-FoxO3a pathway, autophagy. Because immobilization does not change any of the autophagy markers studied, we propose that autophagy is not a regulator of long-term disuse atrophy. Conversely, denervation led to a significant increase in all pro-autophagy markers examined, as has been previous described in the literature (Fig. 4B) (O’Leary and Hood, 2009; Zhao et al., 2007). It is reasonable to speculate that this pronounced increase in autophagy has such a detrimental effect on the muscle that it is interfering with the ability of ActRIIB to prevent denervation atrophy.

Because Akt and SGK can regulate the pro-growth mTOR pathway, we also examined whether this pathway was dysregulated in our models of atrophy. We found that, although denervation does cause a substantial increase in most components of the mTOR complexes, levels of these proteins were all unchanged in immobilized muscle compared with controls (Fig. 5A). We did observe, however, that both atrophies bring about a loss of activity of the mTORC1 substrate p70S6k. Although that is suggestive that both immobilization and denervation lead to decreased protein synthesis, this is brought about through different mechanisms in the two atrophies. Immobilization leads to a loss p70S6k expression, possibly mediated by the loss of the scaffold protein eIF3f (Fig. 5B and supplementary material Fig. S2) (Csibi et al., 2009; Csibi et al., 2008), but denervation resulted in a loss of p70S6k activation – possibly due to an increase in ULK1 activity (Fig. 5B and supplementary material Fig. S3) (Egan et al., 2011; Lee et al., 2007).

A recent report demonstrated that treating a laminin-deficient mouse model with an mTOR inhibitor reduced autophagy and significantly improved the aberrant muscle phenotype of the mice (Ramos et al., 2012). Similarly, we also treated our denervated mouse model with the mTOR inhibitor rapamycin to test whether the upregulation of mTOR that we observed is a compensatory mechanism or is contributing to the atrophy phenotype. We found that, compared with placebo-treated mice, rapamycin had no effect on the denervation atrophy phenotype despite downregulating mTORC signaling in the atrophic muscle (Fig. 6A,B).

Of great interest were our findings that rictor, more so than raptor, is sensitive to rapamycin treatment in our denervation model (Fig. 6C). This complements several reports suggesting that examination of both the mTORC1 and mTORC2 complexes is necessary to fully understand the *in vivo* effects of rapamycin (Lamming et al., 2012; Sarbassov et al., 2006; Ye et al., 2012). We found this to be the case in our models, because rapamycin completely prevented the denervation-induced upregulation of rictor. In addition, the mTORC2 substrates Akt and SGK also exhibited reduced activation with rapamycin treatment compared with placebo-treated denervated muscle. In fact, when compared with sham-operated controls, rapamycin-treated denervated mice demonstrated no significant upregulation in active pAkt and SGK (Fig. 7A). Why there is an upregulation of so many pro-growth proteins even when they are not actually beneficial is still unclear, although finding the reason behind that would likely aid our understanding of how to effectively treat denervation atrophy.

Previous research has indicated that several different pathways can contribute to atrophy pathogenesis. For example, upregulation of PGC-1α has been shown to be important for preventing sarcopenic and ALS-induced muscle atrophy, and activation of the IL-6–STAT3 pathway has been linked to the muscle wasting seen in cancer cachexia (Bonetto et al., 2012; Bonetto et al., 2011; Da Cruz et al., 2012; Wenz et al., 2009). However, it should be noted that the vast majority of papers indicate that it is the IGF-Akt-FoxO3a pathway that is the most important for the progression of muscle atrophy (Lecker and Goldberg, 2002; Lecker et al., 2004; Sacheck et al., 2007). Although it is well-established that Akt can play a substantial role in muscle hypertrophy and atrophy under certain circumstances, it is becoming clear that denervation is an exception to this rule. Even though some evidence has indicated that increasing Akt activation might be beneficial to the muscle during early stages of denervation atrophy (Bodine et al., 2001b), our results nonetheless outline an important point for future treatment approaches – Akt, mTOR and SGK do not represent promising therapeutic targets for individuals suffering from prolonged denervation. A detailed timecourse assessment of the molecular events that occur as denervation atrophy progresses could clarify how certain proteins and pathways are involved in the pathogenesis. Indeed, we are currently in the process of conducting a cohesive study looking at these various signaling pathways at multiple time points after denervation.

In conclusion, our studies bring two very significant contributions to the field of skeletal muscle atrophy. First, disuse atrophy, but not denervation atrophy, can be prevented by the application of a myostatin inhibitor. Myostatin inhibitors are currently being tested in clinical trials and will soon be available for use by physicians. The knowledge that myostatin inhibition is not a ubiquitous treatment option for all forms of atrophy will aid in providing targeted therapies to individual patients. Secondly, denervation atrophy is independent from Akt, mTOR and SGK activation, and therefore does not follow the usual molecular pathway paradigm for muscle wasting. Our study highlights the fundamental differences in the pathophysiologies of disuse and denervation atrophy, and emphasizes the importance of developing unique strategies for their treatment.

## MATERIALS AND METHODS

### Animal models

Mice were housed in facilities in accordance with the Animal Care and Use Committee of Johns Hopkins University School of Medicine safety protocols. All animals used for experiments were 2-month-old male, C57BL/6 mice (Jackson Laboratories, Bar Harbor, ME, USA). Mice were immobilized by use of a surgical staple (Autosuture Royal 35W stapler, Mansfield, MA, USA) attached to the hind foot held against the tibia as previously described (Caron et al., 2009). Mice were denervated by surgical removal of ~6 mm of sciatic nerve from one hindlimb. Control mice were sham operated in the same way, but without the removal of the nerve. Wounds were closed with surgical staples. The mice were monitored for infection, then anesthetized with isoflurane and euthanized after 3 weeks. Skeletal muscles were dissected and either flash-frozen in liquid nitrogen or imbedded in optimal cutting temperature (OCT) compound (Tissue-Tek, Torrance, CA, USA) for cryostat sectioning. Experiments were repeated at least twice with a minimum of five mice per treatment group. Data shown are averages across all experimental sets. All TA mass measurements are normalized to tibia length.

### Drug delivery

Mice were given intraperitoneal injections of soluble ActRIIB at a dosage of 10 mg/kg body weight on days 1, 4, 8 and 15 in a volume of 500 μl PBS. Control and placebo mice were injected with 500 μl PBS on the same days. Soluble ActRIIB was obtained from Dr Se-Jin Lee and was produced as previous described (Lee et al., 2005). Rapamycin (LC Laboratories, Woburn, MA, USA) was dissolved in DMSO and mice were given daily intraperitoneal injections at a dosage of 2 mg/kg or 10 mg/kg body weight diluted to 200 μl in saline solution (saline with 0.2% carboxymethylcellulose and 0.25% Tween80). Control and placebo mice received an equal volume of DMSO diluted to 200 μl in saline solution.

### Sectioning and staining

Tissue imbedded in OCT was sectioned with a cryostat (Microm HM 550) into 10-mm sections. Slides were stained by blocking in 5% BSA/PBS then incubated overnight with primary antibody against laminin-γ1 (Chemicon, Temecula, CA, USA) in 1% BSA/PBS at 4°C. The slides were washed three times for 5 minutes with 1% BSA/PBS, incubated 90 minutes at room temperature with appropriate secondary antibody (Alexa Fluor 488, Invitrogen, Grand Island, NY, USA), washed again three times for 5 minutes with 1% BSA/PBS, and finally mounted with hard set mounting media (Vector Laboratories, Burlingame, CA, USA). Immunofluorescent pictures were taken with an Eclipse i80 microscope (Nikon, Melville, NY, USA) at 10× magnification for myofiber measurement analysis. Myofiber size was determined by measuring the minimum feret diameter (MFD) of 700–1000 fibers for three to five mice per treatment group using Nikon NS elements 2.0 software. Representative pictures of myofiber size were taken at 20× magnification.

### Western blots

Flash-frozen tissue was homogenized using a polytron in Total Protein Extraction with protease inhibitors (Millipore, Billerica, MA, USA) and supplemented with phosphatase inhibitors (Roche, San Francisco, CA, USA). Protein concentration was obtained using the Pierce BCA protein assay (Thermo Scientific, Rockford, IL, USA). 20 μg of total protein lysates were separated on 4–12% gradient Bis-Tris midi or 18% Tris-Glycine mini gels (Invitrogen, Grand Island, NY, USA) then wet transferred to nitrocellulose membrane. Nitrocellulose membranes were blocked for 30 minutes at room temperature in either 5% BSA/PBST or 5% milk/PBST then incubated with primary antibodies overnight at 4°C. Membranes were then washed twice for 20 minutes in PBST, incubated for 1 hour at room temperature with appropriate secondary antibody, and then washed twice for 20 minutes using PBST. Membranes were then developed with SuperSignal West Femto or Dura (Thermo Scientific, Rockford, IL, USA) and images were obtained by exposing membranes to X-ray films. Quantification of all western blots was performed using ImageJ (National Institutes of Health, Bethesda, MD, USA) and GAPDH was used as a loading control to normalize signal intensity. Al antibodies were tested by western blot multiple times for each sample set – figure images with a line indicate where intervening lanes have been removed from a single image to show the most representative band for that treatment group.

### Antibodies

Antibodies were obtained from Cell Signaling, Boston, MA, USA (Smad2 #5339, pERK1/2^T202/Y204^, ERK1/2, pFoxO3a^T32^, p-mTOR^S2448^, mTOR, rictor, raptor, GβL, p4E-BP1^S65^, 4E-BP1, pAkt^S473^, Akt, ATG7, LC3b, SGK, pULK1^S757^), Novus Biologicals, Littleton, CO, USA (eIf3f Lamp2), Epitomics, Burlingame, CA, USA (pSmad3^S423/S425^, Smad3), Invitrogen, Grand Island, NY, USA (pSmad2^S465/S467^), Abcam, Cambridge, MA, USA (p70S6k, p-p70S6k^T389^, Myogenin, FoxO3a, p62, ULK1), Millipore, Billerica, MA, USA (pFoxO3a^S252^, p21), ECM Biosciences, Versailles, KY, USA (atrogin-1), and Santa Cruz Biotechnology, Dallas, TX, USA (GAPDH). Secondary antibodies were obtained from GE Healthcare, Pittsburgh, PA, USA (anti-rabbit, anti-mouse, anti-rat) and Sigma-Aldrich, St Louis, MO, USA (anti-goat).

### Statistical analysis

All figures represent a comparison of control, placebo immobilized/denervated, and treated immobilized/denervated mice. All figure graphs are expressed as means ± s.e.m. and plotted using Excel. Statistical analyses were done using R statistical software to generate a one-way ANOVA followed by Tukey’s HSD pairwise comparison for *P*-values. *P*-value ≤ 5.0×10^−2^ was considered significant.

## Supplementary Material

Supplementary Material
