# Supplementary material for: Denervation atrophy is independent from Akt and mTOR activation and is not rescued by myostatin inhibition
Source: Dis Model Mech. 2014 Feb 6;7(4):471–81. doi: 10.1242/dmm.014126 (PMC3974457; doi:10.1242/dmm.014126)
Supplement: Supplementary Material [file supp_7_4_471__index.html]

Denervation atrophy is independent from Akt and mTOR activation and is not rescued by myostatin inhibition — Supplementary Material 

# Denervation atrophy is independent from Akt and mTOR activation and is not rescued by myostatin inhibition

## DMM014126 Supplementary Material

**Files in this Data Supplement:**

- **Supplementary Material**
