# Supplementary material for: Denervation atrophy is independent from Akt and mTOR activation and is not rescued by myostatin inhibition
Source: Dis Model Mech. 2014 Feb 6;7(4):471–81. doi: 10.1242/dmm.014126 (PMC3974457; doi:10.1242/dmm.014126)
Supplement: Supplementary Material [file supp_7.4.471_DMM014126.pdf]

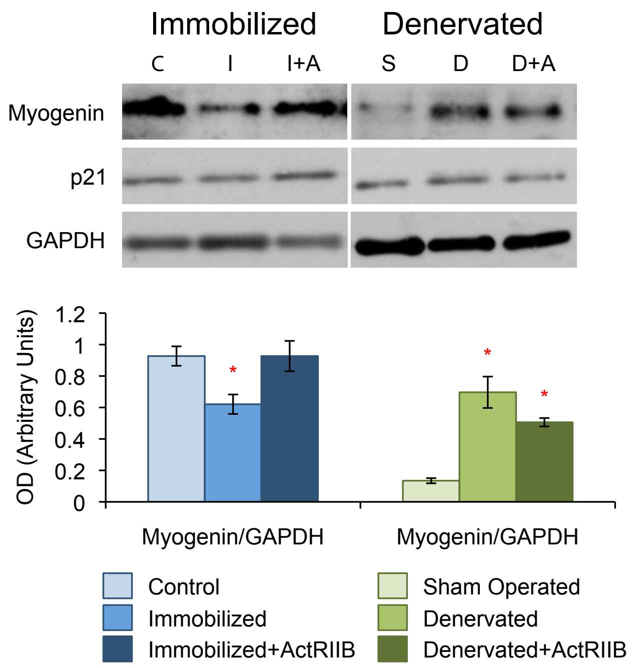

Supplementary Fig. 1. ***Only certain myostatin targets are affected by ActRIIB treatment.*** Western blot analysis of TA muscle protein lysates. Compared to controls (C), immobilization (I) lead to a decrease in myogenin expression but not in mice treated with ActRIIB (I+A). Denervation (D) lead to a substantial increase in myogenin expression compared to sham operated controls (S). However, ActRIIB treatment of denervated mice (D+A) did not lead to further changes in myogenin expression. No change was seen in p21 expression in either the immobilization or denervation models with ActRIIB treatment. Quantitative analysis of blots is displayed in the graph (below) with arbitrary units of mean±s.e.m. *P<5.0x10^{-2} with respect to controls.

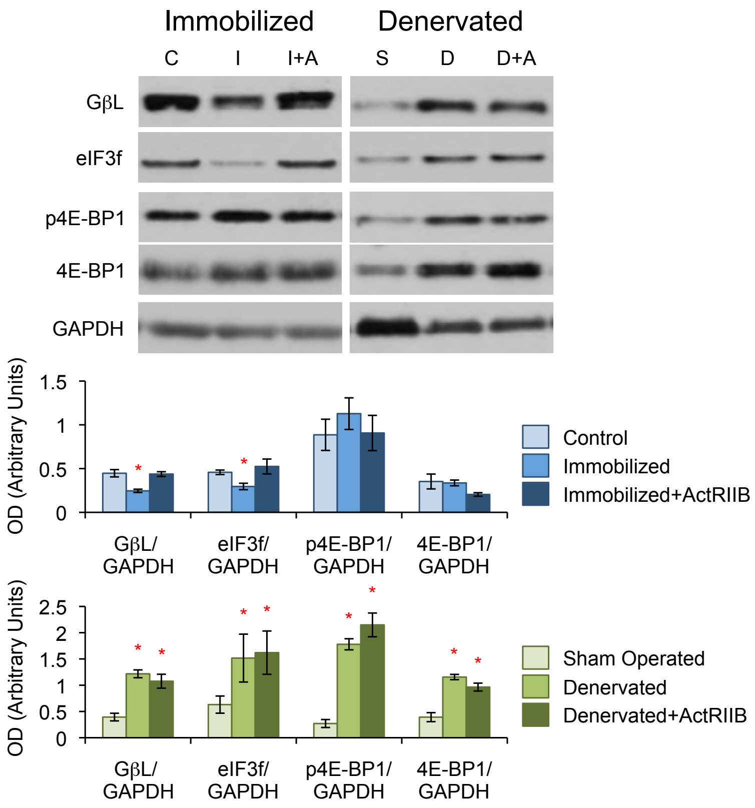

Supplementary Fig. 2. ***Dysregulation of mTOR complex scaffold proteins with disuse and denervation atrophy.*** Western blot analysis of TA muscle protein lysates. Immobilization (I) results in a significant decrease of the mTOR complex scaffold proteins GβL and eIF3f when compared to controls (C), but not when mice are treated with ActRIIB (I+A). No change is seen in the mTORC1 complex substrate 4E-BP1 in immobilized placebo or ActRIIB treated mice. Denervated (D) and ActRIIB treated denervated (D+A) mice had a significant increase in GβL and eIF3f compared to sham operated controls (S). Phosphorylation and total expression levels of 4E-BP1 were increased in denervated muscle with or without ActRIIB treatment. Quantitative analysis of blots is displayed in the graphs (below) with arbitrary units of mean±s.e.m. *$P<5.0\times10^{-2}$ with respect to controls.

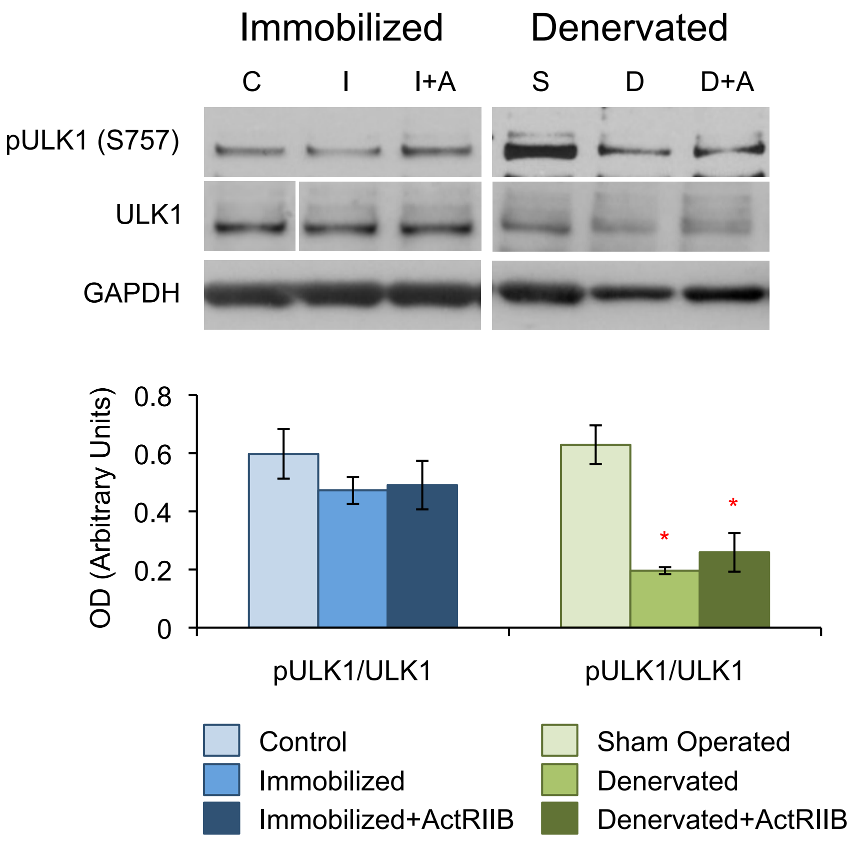

Supplementary Fig. 3. ***Loss of ULK1 inhibitory phosphorylation with denervation, but not disuse, atrophy.*** Western blot analysis of TA muscle protein lysates. Immobilized placebo (I) and ActRIIB treated (I+A) muscle did not show changes in phosphorylation or expression levels of ULK1 when compared to controls (C). Denervation (D) resulted in a decrease in phosphorylation at the inhibitory S757 site of ULK1 when compared to sham operated controls (S). ActRIIB treatment of denervated mice (D+A) did not prevent the loss of phosphorylation. No change was seen in total protein expression of ULK1 in denervated placebo or ActRIIB treated muscle. Quantitative analysis of blots is displayed in the graphs (below) with arbitrary units of mean±s.e.m. $*P<5.0\times10^{-3}$ with respect to controls. Lines indicate where intervening lanes have been removed from a single image to show the most representative band for that treatment group.

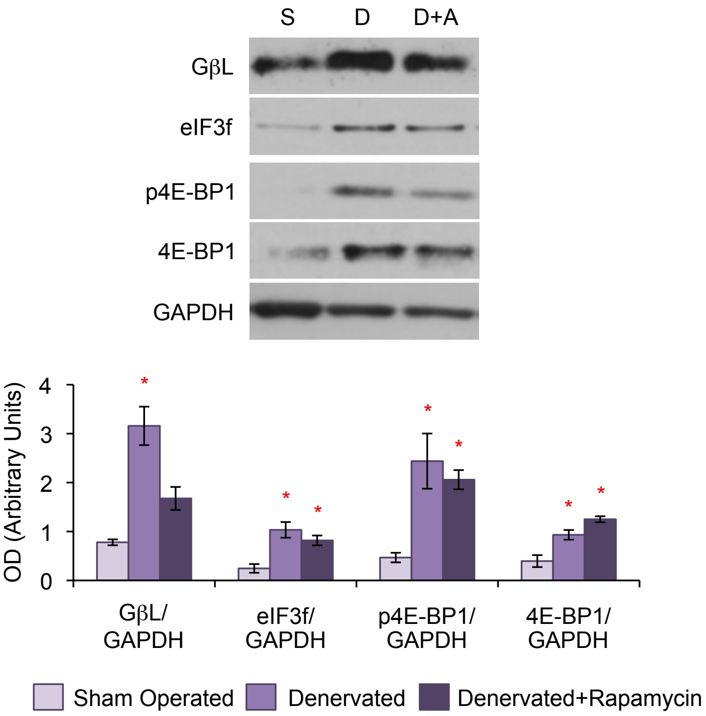

Supplementary Fig. 4. ***Rapamycin treatment of denervated muscle downregulates some components of the mTOR pathway.*** Western blot analysis of TA muscle protein lysates. Denervation (D) lead to an increase in the scaffold proteins GβL and eIF3f when compared to sham operated controls (S). The phosphorylation and expression of 4E-BP1 was also significantly upregulated with denervation. Denervated mice treated with rapamycin (D+R) also showed a substantial increase in GβL but it did not reach significance compared to controls. Rapamycin treatment did not alter eIF3f or 4E-BP1 phosphorylation and expression levels compared to placebo treated denervated muscle. Quantitative analysis of blots is displayed in the graph (below) with arbitrary units of mean±s.e.m. *P<5.0x10^{-2} with respect to controls.

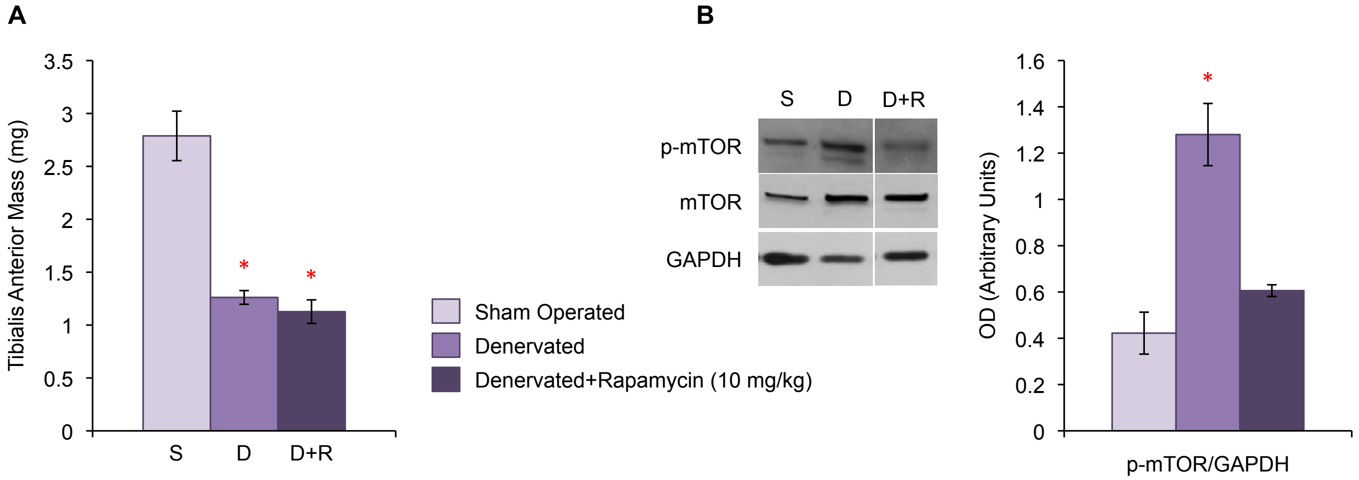

Supplementary Fig. 5. ***A high dose of rapamycin completely inhibits p-mTOR but does not rescue the phenotype.*** (a) Denervation alone (D) and with high dose (10 mg/kg) rapamycin treatment (D+R) resulted in a significant loss of TA mass when compared to sham operated controls (S). (b) Western blot analysis of TA muscle protein lysate. Denervated mice showed a substantial increase in p-mTOR when compared to controls, but not when the mice received high dose rapamycin treatment. Quantitative analysis of blots is displayed in the graph with arbitrary units of mean±s.e.m. For both graphs *P<5.0x10^{-3} with respect to controls.
